# *g*-B_3_N_3_C: a novel two-dimensional graphite-like material

**DOI:** 10.1186/1556-276X-7-624

**Published:** 2012-11-13

**Authors:** Jinyun Li, Daqiang Gao, Xiaoning Niu, Mingsu Si, Desheng Xue

**Affiliations:** 1 Key Laboratory for Magnetism and Magnetic Materials of the Ministry of Education, Lanzhou University, Lanzhou, 730000, China

**Keywords:** *g*-B_3_N_3_C, Graphene, First-principles

## Abstract

A novel crystalline structure of hybrid monolayer hexagonal boron nitride (BN) and graphene is predicted by means of the first-principles calculations. This material can be derived via boron or nitrogen atoms which are substituted by carbon atoms evenly in the graphitic BN with vacancies. The corresponding structure is constructed from a BN hexagonal ring linking an additional carbon atom. The unit cell is composed of seven atoms, three of which are boron atoms, three are nitrogen atoms, and one is a carbon atom. It shows a similar space structure as graphene, which is thus coined as *g*-B_3_N_3_C. Two stable topological types associated with the carbon bond formation, i.e., C-N or C-B bonds, are identified. Interestingly, distinct ground states of each type, depending on C-N or C-B bonds, and electronic bandgap as well as magnetic properties within this material have been studied systematically. Our work demonstrates a practical and efficient access to electronic properties of two-dimensional nanostructures, providing an approach to tackling open fundamental questions in bandgap-engineered devices and spintronics.

## Background

Two-dimensional (2D) nanomaterials, such as graphene and monolayer hexagonal boron nitride (h-BN), are expected to play a key role in future nanotechnology as well as to provide potential applications in next-generation electronics. Recently, novel hybrid structures consisting of a patchwork of BN and C nanodomains (BNC) were synthesized through the use of a thermal catalytic chemical vapor deposition method [1]. This finding immediately has attracted a great deal of research interest [2-4], given that it demonstrates a hitherto efficient route to tune the bandgaps of these 2D materials.

It is well known that the perfect hexagonal and planar structure of BNC largely depends on the good matching between BN and C domains. However, it is indeed an outstanding challenge as BN and C phases are naturally immiscible in 2D [1]. This explains why Ci et al. [1] could observe some wrinkles in the atomic force microscopy image. Such mutual contradiction mainly originates from the domain boundary effect and the staggered potentials of B and N atoms in BNC, which doubtlessly affects their continuous tunable electronic energy gaps. It has been confirmed in the related theoretical calculations [5-9] where the bandgaps show a strong oscillation feature.

The X-ray photoelectron spectroscopy of BNC measured in the work of Ci et al. [1] shows two additional types of C-bonding configurations, which correspond to the C-B and C-N bonds with the bonding energies of around 188.4 and 398.1 eV, respectively. This feature means that two inequivalent C-bonding types, i.e., C-B or C-N bonds, must be present in the boundaries of the hybridized BN and C domains, which have a significant effect on determining the local structures and subsequently vary the electronic properties in this system. For example, the transport channels show a robust characteristic gap when the topological index changes the sign of the valley Hall effect [3]. In addition, both Raman D band at 1,360 cm^−1^ and D’ band at 1,620 cm^−1^are also observed in BNC [1], which were attributed to the lattice disorder or the finite crystal size. This lattice disorder effect might directly introduce vacancies to this 2D hexagonal system [10-12], which is also true in BNC as shown in Figure two (a) of [1].

For the planar BNC structure, although the larger domains would be preferred to decrease the total domain interfacial energy, the randomly distributed hybrid domains and the immiscible phases, as well as the induced vacancies, must result in various complex structures of BNC [13-16]. An immediate consequence is the largely inaccessible synthesis of expected BNC in experiments [17], which next hinders realization of bandgap-engineered applications in actual devices. In this endeavour, exploring the structures and electronic properties associated with C bond formation in BNC which contains C-N or C-B bonds is an interesting topic that must be addressed before widespread synthetic applications. Thus, a simple model of BNC where the C bonds play a crucial role must be considered again. More importantly, a deep theoretical understanding, which originally was concealed behind the complex hybridized structures, is imperative.

**Figure 1:**
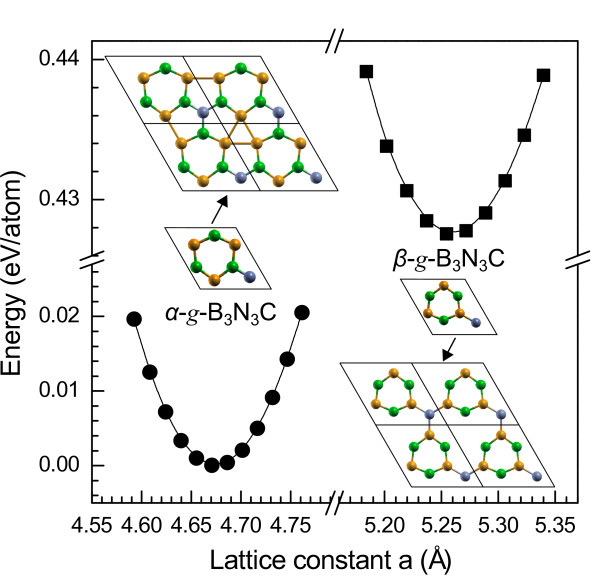
**The total energy per atom as a function of lattice constant*a*for*g*-B**_**3**_**N**_**3**_**C.** Yellow, green, and gray balls represent B, N, and C atoms, respectively. Their respective 2×2 supercells are also given nearby.

Here, we report that such a simple model of BNC may be just the graphitic B_3_N_3_C (*g*-B_3_N_3_C), which is a perfect 2D monolayer graphite-like structure as shown in Figure 1. As mentioned above, the C atom can bond to B and N atoms. Therefore, two topological types of *g*-B_3_N_3_C are easily deduced. One is the *α*-*g*-B_3_N_3_C which is related to the the higher bonding energy of the C-N bond, while the other one is the *β*-*g*-B_3_N_3_C which is constructed based on the lower bonding energy of the C-B bond. It can be seen that such a material is essentially a C-doped graphitic BN (g-BN) with vacancies [11]. The substitution of the N/B atom with a C atom in g-BN with the B/N vacancy will yield the *α*/*β*-g-B_3_N_3_C structure. The interactions among the C atoms and/or vacancies as well as the C-bonding types (C-B and C-N bonds) in *g*-B_3_N_3_C significantly alter its electronic properties. To explore this effect, standard density functional theory with different functional (see the following discussion) calculations has been carried out for this predicted material. Remarkably, such material displays two distinct electronic structure properties: *α*-*g*-B_3_N_3_C is a semiconductor, while *β*-*g*-B_3_N_3_C behaves like a metal and leads to a magnetic ground state.

This paper is arranged as follows: In the second section, we present the computational method used in this work, followed by the electronic bandgap in *α*-*g*-B_3_N_3_C and magnetism in *β*-*g*-B_3_N_3_C in the third section. We then conclude this paper in the fourth section.

## Methods

For structural optimization, we employed density functional theory with the generalized gradient approximation (GGA) of Perdew-Burke-Ernzerhof (PBE) [18] for the exchange-correlation (XC) potential within the projector augmented wave method as implemented in VASP [19,20]. An all-electron description, the projector augmented wave method, is used to describe the electron-ion interaction. The cutoff energy for plane waves is set to be 500 eV, and the vacuum space is at least 15 Å, which is large enough to avoid the interaction between periodical images. A 7×7×1 Monkhorst-Pack grid is used for the sampling of the Brillouin zone during geometry optimization. All the atoms in the unit cell were allowed to relax, and the convergence of force is set to 0.01 eV/Å. Additionally, spin polarization is turned on during the relaxation processes. All other calculations of accurate electronic properties were performed using the full-potential linearized augmented plane-wave [21] method as implemented in the WIEN2k code [22]. It is well known that different XC potentials can lead, depending on the studied materials and properties, to results which are in very bad agreement with the experiment, e.g., for the bandgap of semiconductors and insulators which is severely underestimated or even absent [23]. For this reason, the modified Becke-Johnson (MBJ) [24,25] potential in the framework of local-density approximation (LDA) [26,27] is taken to calculate the bandgap of *α*-*g*-B_3_N_3_C, while the magnetism in *β*-*g*-B_3_N_3_C is described by the PBE (the standard GGA for materials) potential.

## Results and discussion

### Electronic bandgap in *α*-*g*-B_3_N_3_C

Figure 1 plots the total energy per atom against the lattice constant *a* (the lattice constant *c* is fixed) for *g*-B_3_N_3_C. It can be seen that the total energy of *g*-B_3_N_3_C as a function of lattice constant *a* has a single minimum, meaning that the geometrical structure would be stable. Particularly, the charge population analysis reveals that the electron density around the C-N bond in *α*-*g*-B_3_N_3_C is much higher than that around the C-B bond in *β*-*g*-B_3_N_3_C, showing that the C-N bond is relatively strong, which is also consistent with the experimental results [1]. This strong interaction between C and N atoms in *α*-*g*-B_3_N_3_C directly results in a short C-N bond length (see the following paragraph) and can balance the strain of the monolayer graphite-like structure. Thereby, *α*-*g*-B_3_N_3_C could be a more thermodynamically stable topological phase against *β*-*g*-B_3_N_3_C, which has a lower total energy of around 0.43 eV/atom compared with *β*-*g*-B_3_N_3_C.

**Figure 2:**
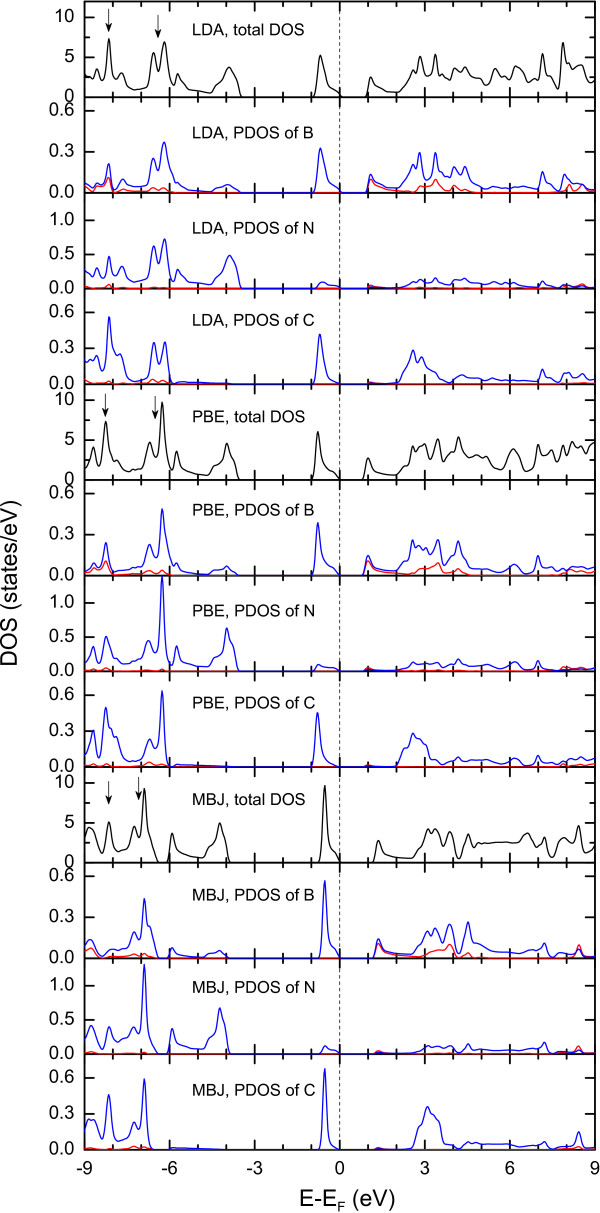
**Total and partial DOS of*α*-*g*-B**_**3**_**N**_**3**_**C for three XC potentials LDA, PBE, and MBJ.** The vertical dotted line denotes the Fermi level and also indicates the end of the fundamental bandgap which starts at *E* − *E*_F_ = 0eV. The black, blue, and red lines correspond to the total, *p*, and *s* DOS, respectively.

To explore further the mechanical stability of *α*-*g*-B_3_N_3_C, the optimized lattice constant *a* = 4.67Å is first obtained, as depicted in the left panel of Figure 1. Importantly, the C-N bond length converged to 1.31 Å, which is considerably reduced from the typical bond lengths of 1.37 to 1.48 Å in the related materials [28]. This strongly suggests the nature of the higher binding energy of the C-N bond [1], which also influences the B-N bonding and extends its length. The obtained B-N bond length is 1.48 Å, which is slightly bigger than the value of 1.45 Å in h-BN. The calculated partial density of states (PDOS) is shown in Figure 2. The valence band is dominated by B *p* and C *p* states, while the conduction band is only dominated by B *p* states. There, one can find the majority of C *p* states to be semicore, lying 6 to 9 eV below the Fermi level. These states interact with those comprising the valence band with the same symmetry. As a result, there is a small admixture of C *p* and N *p* states close to the Fermi level. However, in *α*-*g*-B_3_N_3_C, one finds significant admixture of C *p* and N *p* states in the semicore energy window with 6 to 9 eV below the Fermi level. This suggests that the C *p*-N *p* interaction in the semicore region contribution to the C-N bonding is significantly more important in *α*-*g*-B_3_N_3_C than the C *p*-N *p* interaction close to the Fermi level.

**Figure 3:**
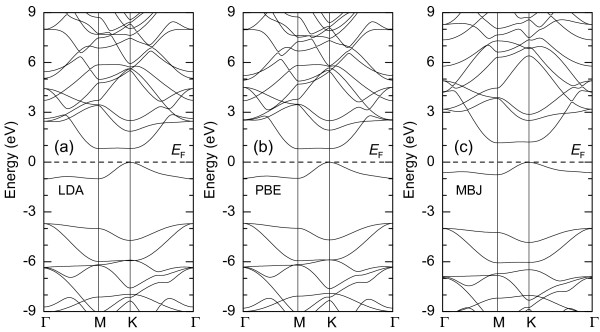
**Calculated band structures for *α*-*g*-B**_**3**_**N**_**3**_**C model with the three XC potentials.(a)** LDA XC potential. **(b)** PBE XC potential. **(c)** MBJ XC potential.

Now, let us look at the band structures of *α*-*g*-B_3_N_3_C, as given in Figure 3. It explicitly demonstrates that all three XC potentials give similar band structures. The highest occupied crystalline orbitals are located at the K point of the reciprocal space, while the lowest unoccupied crystalline orbitals appear at the M point. This leads to an indirect bandgap semiconductor. To obtain the bandgap more accurately (see Figure 3c), the MBJ XC functional is used [24,25]. The bandgap of *α*-*g*-B_3_N_3_C within MBJ is obtained to be 1.22 eV, which nicely locates the middle region between 0.59 and 1.80 eV for the BNC samples with 12.5% and 50% C contents, respectively [4]. Note that the C content in *α*-*g*-B_3_N_3_C is 25%. In the experiments [1], the absorption edges are redshifted as the C concentrations increase, which shows a tunable mechanism of an optical bandgap in actual applications. By comparing with [1] where BNC with around 65% C concentration shows an optical bandgap of 1.62 eV, we infer that such a higher energy absorption edge (take into account the bandgap of 1.22 eV in our case with 25% C concentration) arises from the formation of individual BN and graphene domains. In this way, the even distribution of C in BNC systems might serve as a good guide to find alternative solutions to existing bandgap-engineered applications. Future research can test this prediction directly.

In addition, the bandgaps based on the LDA and PBE methods are equal to 0.83 eV (see Figure 3a,b). The relative bandgap correction from MBJ with respect to LDA, $R\;=\;(\Delta_{\text{MBJ}}-\Delta_{\text{LDA}})/\Delta_{\text{MBJ}}$ with *Δ*_MBJ/LDA_ being the bandgap, is about 32%. We can see that our calculated R for *α*-*g*-B_3_N_3_C ( shows an excellent agreement with the 16 *sp* semiconductors) lies within the range of 16.0% to 100.0% (see Table 1 for details). The correction value is particularly very close to that in BN (25%), GaN (42%), AlP (37%), and AlN (25%). This finding is not surprising because the listed four solids have at least one element close to that of *α*-*g*-B_3_N_3_C in the periodic table of elements. It means that the similar chemical circumstances in these materials can be well described by the same XC functionals. This underlyingly confirms our prediction validity and that the *α*-*g*-B_3_N_3_C might be carried out experimentally.

**Table 1:** The bandgaps and the relative bandgap correction R of 16 *sp* semiconductors and the predicted *α*-*g*-B_3_N_3_C

**Solid**	**LDA**	**MBJ**	R	**Expt.**
C	4.11	4.93	16.6	5.48
Si	0.47	1.17	59.8	1.17
Ge	0.00	0.85	100.0	0.74
LiF	8.94	12.94	30.9	14.20
LiCl	6.06	8.64	29.9	9.40
MgO	4.70	7.17	34.4	7.83
ScN	−0.14	0.90	115.6	0.90
SiC	1.35	2.28	40.8	2.40
BN	4.39	5.85	25.0	6.25
GaN	1.63	2.81	42.0	3.20
GaAs	0.30	1.64	81.7	1.52
AlP	1.46	2.32	37.1	2.45
ZnS	1.84	3.66	49.7	3.91
CdS	0.86	2.66	67.7	2.42
AlN	4.17	5.55	24.9	6.28
ZnO	0.75	2.68	72.0	3.44
*α*-*g*-B_3_N_3_C	0.83	1.22	32.0	−

The theoretical and experimental bandgaps (in eV) of the 16 *sp* semiconductors are directly taken from [25]. The relative bandgap correction R (in %) is calculated from the equation $R=(\Delta_{\text{MBJ}}-\Delta_{\text{LDA}})/\Delta_{\text{MBJ}}$, where *Δ*_MBJ_ and *Δ*_LDA_ are the calculated bandgaps using XC potentials MBJ and LDA.

From Figure 2, which shows the DOS of *α*-*g*-B_3_N_3_C, we can see that the effect of the MBJ potential is to shift up (with respect to LDA/PBE) the unoccupied B 2*s* and 2*p* states. Here, three major differences between the LDA/PBE and MBJ methods can be extracted: (a) Another obvious effect of MBJ potentials is to shift down the middle of the valence band at around −4.0 eV. (b) The hybridization of *s* and *p* states of dominant B 2*s* and other atoms’ 2*p* states at the bottom of the valence band is very strong in MBJ calculations (denoted as down arrows in Figure 2). (c) The correction character of *α*-*g*-B_3_N_3_C is much more pronounced with the MBJ than with the LDA/PBE, which narrows the valence band just below the Fermi level. We would like to stress that the MBJ potentials open a bandgap of 0.39 eV in the *α*-*g*-B_3_N_3_C model compared to the result of LDA, which is consistent with the orbital-dependent potential principle [24].

**Figure 4:**
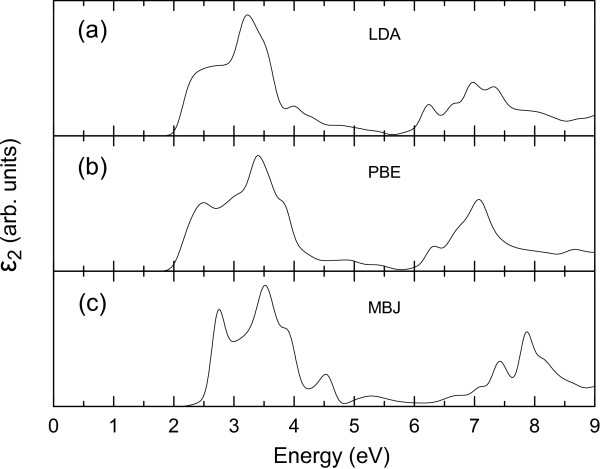
**The optical absorption expressed by the imaginary part of the dielectric tensor*ε***_***2***_***forα-g*-B**_***3***_***N***_***3***_***C.*** The imaginary part of the dielectric tensor is averaged for four different directions. Three XC potentials including **(a)** LDA, **(b)** PBE, and **(c)** MBJ are shown in the calculations.

The optical absorption spectrum for *α*-*g*-B_3_N_3_C within MBJ potentials (see Figure 4c) shows a big absorption packet with two adjacent peaks in the range of 2.5 to 5.0 eV, which originates from the band structure as shown in Figure 3c. As an indirect bandgap material, the transition from the K point to *Γ*point is very weak as the momentum conservation rule is not satisfied here, and thus, the corresponding characteristic photoluminescence in the optical absorption spectrum can be negligible. This is also true in our case for *α*-*g*-B_3_N_3_C. The first peak corresponds to the direct bandgap transition at the M point. The second peak comes from the larger direct gap from higher energy states located at the *Γ* point. More importantly, these two peaks are not separated distinctly. This feature can be attributed to the even distribution of C atoms in *α*-*g*-B_3_N_3_C as mentioned above, which shows a different formation mechanism compared with the hybrid BNC in the experiment [1].

Correction effects were taken into account by adding a LDA correction potential in MBJ [25]. This important physical effect opens an additional bandgap by mimicking very well the behavior of orbital-dependent potentials and causes a rigid blueshift of the absorption spectrum compared with the LDA/PBE curves as shown in Figure 4. This explains the excellent qualitative agreement of the hybrid exchange-potential optical absorption spectrum seen in Figure 4, due to a compensation of significant errors within the standard DFT methods.

### Magnetism in *β*-*g*-B_3_N_3_C

The predicted structure of *β*-*g*-B_3_N_3_C is shown in the right panel of Figure 1. The lattice constant of *β*-*g*-B_3_N_3_C is obtained to be 5.26 Å as shown in Figure 1. The results show the equilibrium value *d*_BC_ = 1.52 Å, which is close to the value of graphite-like BC_3_, which is 1.55 Å [29]. It is to be noticed that all the equilibrium values, *d*_BN_, in *α*-*g*-B_3_N_3_C are equal to 1.42 Å, which is slightly less than the value of 1.45 Å in the pristine BN sheet. This implies the stronger B-N bonds formed in *β*-*g*-B_3_N_3_C. Our calculations show that the *βg*-B_3_N_3_C leads to a ground state with a magnetic moment of 0.68 *μ*_B_. The nonmagnetic state is 0.07 eV higher than this ground state.

**Figure 5:**
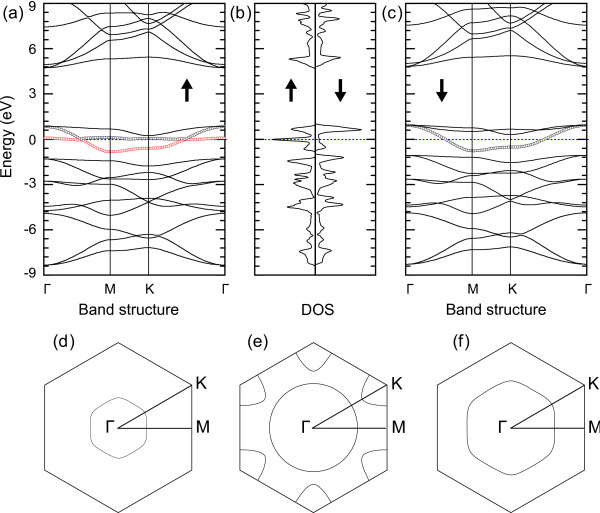
**Band structures, spin-resolved TDOS, and Fermi surfaces for***β***-*g*-B**_**3**_**N**_**3**_**C.(a, b, c)**Band structures and spin-resolved TDOS for *β*-*g*-B_3_N_3_C. The dotted line indicates the Fermi level. The arrow denotes the spin polarization direction: up for spin up and down for spin down. **(d, e, f)** Fermi surfaces drawn in the first Brillouin zone and the corresponding high-symmetry points; **(d)** and **(e)** for spin up and **(f)** for spin down.

**Figure 6:**
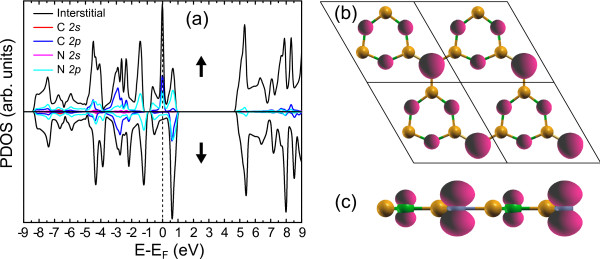
**PDOS and spin density for***β***-*g*-B**_**3**_**N**_**3**_**C.(a)** PDOS on the interstitial region and on the 2*s* and 2*p* orbitals of C and N atoms in *β*-*g*-B_3_N_3_C. **(b)** The 3D iso-surface plot of spin density for the 2×2 supercell at the value of 0.04 e/Å^3^. **(c)** A side view of spin density corresponding to (b).

From the band structures, we see that although both the pristine BN sheet and graphene are nonmagnetic, the *β*-*g*-B_3_N_3_C model can be spin-polarized. It is necessary to discuss magnetism in more detail from its electronic structures. Figure 5a,b,c presents the band structure and spin-resolved total density of state (TDOS). Remarkably, two bands cross the Fermi level (black and red circles in Figure 5a) and make the Fermi energy level occupied completely along the entire high-symmetry lines. In contrast, the spin-down one does not possess such a strongly localized feature, just one band (black circles) crosses the Fermi level monolithically as shown in Figure 5c. The calculated magnetic moment in *β*-*g*-B_3_N_3_C should originate from this asymmetric spin-dependent localization. The corresponding strong spin splitting can be further confirmed from TDOS as shown in Figure 5b. In addition, close examination of the top valence bands (see Figure 6a) indicates that the strong localization mainly comes from the 2*p* atomic orbitals of C and N atoms. Here, we also show the Fermi surfaces of *β*-*g*-B_3_N_3_C in the first Brillouin zone. The unique feature of the Fermi surface which is almost parallel to the high-symmetry lines (see Figure 5d,e,f) is the direct manifestation of the bands near the Fermi level in Figure 5a,c.

It should be noticed that such magnetism is induced without transition metals and without external perturbations so that *β*-*g*-B_3_N_3_C behaves as the first, theoretically predicted, metal-free magnetic material in the hybrid BNC system. Apparently, our finding points out a new direction for further related experimental investigations in spintronics.

We now address the possibility of the origin of magnetism in *β*-*g*-B_3_N_3_C. Based on the orbital-resolved density of state as shown in Figure 6a, the magnetic moment is mainly ascribed to the 2*p* orbitals of C and three N atoms. The spin polarization of C atom offers a magnetic moment of 0.18 *μ*_B_. A total magnetic moment of 0.18 *μ*_B_ is shared equally by the 2*p* orbitals of three N atoms. The remaining magnetic moments distribute evenly in the interstitial region among N atoms. This is conceivable because *βg*-B_3_N_3_C, if compared with other BNC systems, has large interspaces between N atoms (see the 2×2 supercell in Figure 1 or Figure 6b). The remarkable feature is that the equilibrium surface density of *β*-*g*-B_3_N_3_C is around 1.27 times larger than that of *α*-*g*-B_3_N_3_C. Owing to the large interspaces between atoms, the hydrogen storage in *β*-*g*-B_3_N_3_C may be expected [30]. The detailed description of hydrogen storage related to *β*-*g*-B_3_N_3_C is beyond the scope of this work.

Concerning the special carbon atom in *β*-*g*-B_3_N_3_C, the counting of four valence electrons is as follows: Three electrons participate in the *sp*^2^ hybrid orbital, which forms a planar structure. The remaining one electron is then redistributed in the whole unit cell due to the enhanced B-N covalent bond (with shorter bond length compared with the value in pristine h-BN), which makes the magnetic properties more complicated. From the fourth electron, only 18% of the electron still fills the *Π*-orbital of the C atom and contributes a magnetic moment of 0.18 *μ*_B_. This is in fairly good agreement with theoretical description of the *Π*-orbital state to the local magnetic moment of approximately 0.3 *μ*_B_in the graphene-like systems [31,32]. Around 32% of the fourth electron mainly resides at the interstitial region, which plays a crucial role in *βg*-B_3_N_3_C as follows: (a) enhances the B-N covalent bond, (b) provides the main interstitial magnetic moment of 0.32 *μ*_B_, and (c) promotes the 2*p*_*z*_of the N atom spin-polarized slightly with a magnetic moment of 0.06 *μ*_B_per N atom. The remaining percentage of the fourth electron acts as the conduction electrons and makes the system metallic, which dominates the mechanism of ferromagnetic ordering in *βg*-B_3_N_3_C. In the case of *βg*-B_3_N_3_C, we can see that the electron spin at the localized *Π*-orbital state of C and N atoms as well as the interstitial region compels two energy bands localized strictly along the entire high-symmetry lines, i.e., the *Γ*-M-K-*Γ*line (see Figure 5a). Thus, the RKKY interaction [10,33] among the magnetic sites through the residual conduction electrons forms a spin ordering in these orbitals, which is the physical origin of ferromagnetism in *βg*-B_3_N_3_C. Figure 6b,c respectively plot the top and side views of the 3D iso-surfaces for net magnetic charge density in the *xy* plane. This finding is insightful, and three major points deserve comment: (a) The C site is more spin-polarized as compared with each N site. (b) The dumbbell-like magnetic moment distribution along the *z* direction implies that the 2*p*_*z*_ orbital becomes partially filled with one spin-up electron. (c) The induced moments are ferromagnetic coupled between the N and C sites based on the RKKY exchange interaction model as mentioned above.

## Conclusions

In summary, we have predicted a novel crystalline material *g*-B_3_N_3_C, which displays two distinct electronic properties where the selective bonding type of the C atom is a key parameter for future industrial processes. *α*-*g*-B_3_N_3_C is a semiconductor, while *β*-*g*-B_3_N_3_C behaves like a metal and holds a magnetic moment of 0.68 *μ*_B_. Importantly, compared with the hybrid BNC, *g*-B_3_N_3_C is proposed to have a simple structure, which can be applied in various fields due to its unique properties.

## Competing interests

The authors declare that they have no competing interests.

## Authors’ contributions

MSS conceived, designed, and optimized the structure of *g*-B_3_N_3_C. JYL performed the calculations, analyzed the data, and drew the figures. DQG, NXN, and DSX discussed the results. MSS wrote the paper. All authors read and approved the final manuscript.

## References

[B1] Ci L Song L Jin C Jariwala D Wu D Li Y Srivastava A Wang ZF Storr K Balicas L Liu F Ajayan PM Atomic layers of hybridized boron nitride and graphene domains Nat Mater 2010 9 430 435 10.1038/nmat2711 20190771

[B2] Rubio A Hybridized graphene: nanoscale patchworks Nat Mater 2010 9 379 380 10.1038/nmat2746 20414215

[B3] Jung J Qiao Z Niu Q MacDonald AH Transport properties of graphene nanoroads in boron nitride sheets Nano Lett 2012 12 2936 2940 10.1021/nl300610w 22524401

[B4] Bernardi M Palummo M Grossman JC Optoelectronic properties in monolayers of hybridized graphene and hexagonal boron nitride Phys Rev Lett 2012 108 226805 226809 23003640 10.1103/PhysRevLett.108.226805

[B5] Li J Shenoy VB Graphene quantum dots embedded in hexagonal boron nitride sheets Appl Phys Lett 2011 98 013105 013107 10.1063/1.3533804

[B6] Lam K Lu Y Feng YP Liang G Stability and electronic structure of two dimensional Cx(BN)y,compound Appl Phys Lett 2011 98 022101 022103 10.1063/1.3535604

[B7] Seol G Guo J Bandgap opening in boron nitride confined armchair graphene nanoribbon Appl Phys Lett 2011 98 143107 143109 10.1063/1.3571282

[B8] da Rocha Martins J Chacham H Disorder and segregation in B-C-N graphene-type layers and nanotubes: tuning the band gap ACS Nano 2010 5 385 393 21186786 10.1021/nn101809j

[B9] Manna AK Pati SK Tunable electronic and magnetic properties in BxNyCz nanohybrids: effect of domain segregation J Phys Chem C 2011 115 10842 10850 10.1021/jp202195b

[B10] Lehtinen PO Foster AS Ayuela A Krasheninnikov A Nordlund K Nieminen RM Magnetic properties and diffusion of adatoms on a graphene sheet Phys Rev Lett 2003 91 017202 017205 12906568 10.1103/PhysRevLett.91.017202

[B11] Si MS Xue DS Magnetic properties of vacancies in a graphitic boron nitride sheet by first-principles pseudopotential calculations Phys Rev B 2007 75 193409 193412

[B12] Si MS Li JY Shi HG Niu XN Xue DS Divacancies in graphitic boron nitride sheets Europhys Lett 2009 86 46002 46007 10.1209/0295-5075/86/46002

[B13] Pruneda JM Native defects in hybrid C/BN nanostructures by density functional theory calculations Phys Rev B 2012 85 045422 045427

[B14] Mazzoni MSC Nunes RW Azevedo S Chacham H Electronic structure and energetics of BxCyNz layered structures Phys Rev B 2006 73 073108 073111

[B15] Enyashin A Makurin Y Ivanovskii A Quantum chemical study of the electronic structure of new nanotubular systems: α-graphyne-like carbon, boron-nitrogen and boron-carbon-nitrogen nanotubes Carbon 2004 42 2081 2089 10.1016/j.carbon.2004.04.014

[B16] Dutta S Pati SK Half-metallicity in undoped and boron doped graphene nanoribbons in the presence of semilocal exchange-correlation interactions J Phys Chem B 2008 112 1333 1335 10.1021/jp710637c 18189386

[B17] Han W Wu L Zhu Y Watanabe K Taniguchi T Structure of chemically derived mono- and few-atomic-layer boron nitride sheets Appl Phys Lett 2008 93 223103 223105 10.1063/1.3041639

[B18] Perdew JP Burke K Ernzerhof M Generalized gradient approximation made simple Phys Rev Lett 1996 77 3865 3868 10.1103/PhysRevLett.77.3865 10062328

[B19] Kresse G Furthmüller J Efficiency of ab-initio total energy calculations for metals and semiconductors using a plane-wave basis set Comp Mater Sci 1996 6 15 50 10.1016/0927-0256(96)00008-0 9984901

[B20] Kresse G Furthmüller J Efficient iterative schemes for ab initio total-energy calculations using a plane-wave basis set Phys Rev B 1996 54 11169 11186 10.1103/PhysRevB.54.11169 9984901

[B21] Madsen GKH Blaha P Schwarz K Sjöstedt E Nordström L Efficient linearization of the augmented plane-wave method Phys Rev B 2001 64 195134 195142

[B22] Blaha P Schwarz K Madsen GKH Kvasnicka D Luitz J WIEN2k, An Augmented Plane Wave + Local Orbitals Program for Calculating Crystal Properties 2001 Techn. Universität Wien, Austria

[B23] Heyd J Peralta JE Scuseria GE Martin RL Energy band gaps and lattice parameters evaluated with the Heyd-Scuseria-Ernzerhof screened hybrid functional J Chem Phys 2005 123 174101 174108 10.1063/1.2085170 16375511

[B24] Becke AD Johnson ER A simple effective potential for exchange J Chem Phys 2006 124 221101 221104 10.1063/1.2213970 16784253

[B25] Tran F Blaha P Accurate band gaps of semiconductors and insulators with a semilocal exchange-correlation potential Phys Rev Lett 2009 102 226401 226404 19658882 10.1103/PhysRevLett.102.226401

[B26] Kohn W Sham LJ Self-consistent equations including exchange and correlation effects Phys Rev 1965 140 A1133 1138 10.1103/PhysRev.140.A1133

[B27] Perdew JP Wang Y Accurate and simple analytic representation of the electron-gas correlation energy Phys Rev B 1992 45 13244 13249 10.1103/PhysRevB.45.13244 10001404

[B28] Heyrovska R Structures of the molecular components in DNA and RNA with bond lengths interpreted as sums of atomic covalent radii Open Struct Biol J 2008 2 1 7 10.2174/1874199100802010001

[B29] Tomanek D Wentzcovitch RM Louie SG Cohen ML Calculation of electronic and structural properties of BC3 Phys Rev B 1988 37 3134 3136 10.1103/PhysRevB.37.3134 9944901

[B30] Mapasha RE Ukpong AM Chetty N Ab initio studies of hydrogen adatoms on bilayer graphene Phys Rev B 2012 85 205402 205413

[B31] Tada K Haruyama J Yang HX Chshiev M Matsui T Fukuyama H Ferromagnetism in hydrogenated graphene nanopore arrays Phys Rev Lett 2011 107 217203 217207 22181918 10.1103/PhysRevLett.107.217203

[B32] Lee H Son Y Park N Han S Yu J Magnetic ordering at the edges of graphitic fragments: magnetic tail interactions between the edge-localized states Phys Rev B 2005 72 174431 174438

[B33] Bruno P Chappert C Ruderman-Kittel theory of oscillatory interlayer exchange coupling Phys Rev B 1992 46 261 270 10.1103/PhysRevB.46.261 10002208

